# Obstetrics, Gynecology and Pelvic Surgery

**Published:** 1897-08

**Authors:** William Warren Potter

**Affiliations:** Buffalo, N. Y., Examiner in obstetrics, New York State Medical Examining and Licensing Board


### OBSTETRICS, GYNECOLOGY AND PELVIC SURGERY.

CONDUCTED BY WILLIAM WARREN POTTER, M. D., Buffalo, N. Y.,
Examiner in obstetrics, New York State Medical Examining and Licensing Board.

### PUERPERAL ECLAMPSIA.

DR. CHARLES M. ELLIS, of Elkton, Maryland, gave the indications (*Maryland Med. Jour.*) for the necessity of artificial delivery. He called special attention to the great danger of the convulsions of pregnancy before term. His experience included eight cases, occurring at different stages of pregnancy, and showed clearly that when a convulsion occurs before term, unless it is of systemic origin, immediate delivery is imperative, without regard to the presence or absence of uterine contractions or the condition of the os as to dilatation. If the convulsions begin early, the uterus should be emptied by the most expeditious method, and all medicinal treatment should be secondary to this one great object. This is necessary for the reason that the percentage of fatalities from eclampsia is fully 50 per cent.

This high death-rate is greatly exceeded when the delivery is not accomplished, or if it is delayed until several convulsions have occurred, or until uterine contraction and dilatation have supervened.

Of the eight cases seen by the author, five died and three recovered. Of the five that died, premature delivery was effected, one on the sixth day after the initial convulsion, one forty-eight hours after, one eighteen hours after, three others having followed, and the patient being moribund at the time, and one lay in convulsions three days without any attempt at delivery. The experience gained from these eight cases would certainly justify premature delivery.

Dr. Ellis had never seen a death occur before delivery after the operation had been initiated, and he believed the uterus should be evacuated immediately after the first convulsion. When albumen appears in the urine, the more imminent is the danger of eclampsia, and, if this accident is threatened, it may be necessary for the attending physician to hasten delivery without waiting for the convulsive seizure. He denounced the indiscriminate use of morphia hypodermically in cases like these.

---

THE SERUM TREATMENT OF PUERPERAL FEVER.

GAULARD (*Presse Medicale—Fort Wayne Med. Magazine*,) reports two cases of puerperal fever treated by serum. A rickety woman with a contracted pelvis had a protracted labor. The case was one of false presentation. The perineum was torn to the anus, but sutured at once. One week after delivery the temperature rose to 105° F., and remained at that height for three or four days. Gaulard saw her four days later, when the pulse was 140 and irregular, and diarrhea present. The perineal wound was discharging pus and on the vagina there was some sloughs. The uterus was curetted, nothing, however, of importance coming away. Subsequently the uterus was packed with iodoform gauze and the perineum resutured. The next day the temperature fell to 102.7° F., but on the second day it rose again, and her general condition became serious. At this time 10 c. c. of Marmorek's antistreptococcic serum were injected into the abdominal wall. The temperature fell slightly on the following day, and a second injection of two cubic centimeters was given. From this time the temperature fell steadily and the patient made a speedy recovery.

The second case was also a rickety woman. It was her fourth pregnancy. The first labor was natural: in the two others the delivery was effected by forceps. The antero-posterior diameter of the pelvis was three and three-quarter inches. As an unsuccessful attempt had been made outside of the hospital to apply the forceps, a basiotripsy was performed, delivery effected, and a douche of 1-400 bichloride of mercury was given. The temperature rose rapidly and two days after delivery it had risen to 104° F. The uterus was swabbed out with creosoted glycerine, some putrid fragments coming away, and plugged with iodoform gauze. On the fourth day 10 c. c. of antistreptococcic serum were injected, and repeated on the fifth and sixth days. After the third injection the temperature fell to 102.9° F. Another injection was now given, and the temperature fell to 101.5° F., reaching the normal on the eleventh day after delivery. While the temperature was falling, she was seized with bilious vomiting and meteorism, the pulse remaining as before, about 120. The vomiting became uncontrollable, she became comatose, and died on the thirteenth day. Goulard believes that the serum was the cause of the vomiting. He fears that too much serum was injected, for at the autopsy there was no sign of suppuration or peritonitis. The question of maximum dose of serum has yet to be determined. The serum does not do away with the necessity of using the curette, but if the germs have already entered the blood, it may be employed against them and their toxins.

---

### OBJECTIONS TO THE REMOVAL OF THE UTERUS.

Dr. Alex. Hugh Ferguson, Chicago, (*Am. Jour. Surg. and Gynecology,*) objects seriously to removal of the uterus when pus-tubes are the cause of mischief, because: (1) It takes longer to do the operation, and the mortality is higher; (2) the primary hemorrhage is greater and the secondary more liable to ensue; (3) greater shock; (4) more liable to injure ureters, bladder and rectum; (5) the patient is less of a woman, anatomically and socially; (6) the vagina is shortened; (7) hernia of the vagina is liable to follow ; (8) a healthy uterus is often removed on account of the difficulty of diagnosticating, beforehand, the amount of disturbance caused by the inflamed tubes, ovaries and uterus respectively.

### THE INFLUENCE OF CLIMATE ON MENSTRUATION.

JOUBERT, in an article in the *Indian Medical Gazette*, (*Med. and Surg. Reporter*,) says that from a careful study based upon over 3,000 patients, between the ages of ten and nineteen years, he has arrived at the conclusion that the reason why girls in tropical countries menstruate at a relatively earlier age than Europeans is not because of the influence of the climate, but because of too much sexual excitement.

### LACTATION ATROPHY OF THE UTERUS.

VINEBERG (*Amer. Jour. Med. Sc.*, Vol. CXII., No. 1,) contributes a paper to the study of this subject. He has been led to the following conclusions :

1. Modern researches tend to prove that post-puerperal involution consists chiefly in a retraction and contraction of the individual muscle-fibers, whereby the whole uterus is reduced in size.

2. When involution goes on to its full completion the uterus is reduced to a size smaller than that of the nonparous organ.

3. This condition of complete involution is known as post-puerperal hyperinvolution. It is principally seen in nursing women, and from this circumstance has received the cognomen *lactation atrophy*.

4. The so-called lactation atrophy is a normal and desirable condition. It is temporary in its duration, but very rarely under favorable circumstances may become permanent.

5. When the parturient is unable to perform the function of lactation it is the duty of the physician to endeavor to bring about hyperinvolution by other means at his disposal. An observance of this course will prevent many a woman from developing a host of gynecological affections which frequently result from imperfect involution.

### FACE PRESENTATIONS.

P. DREYER (*Norsk Mag. for Laegevidensk.*, No. 3, 1897, abstracted in *Br. Med. Jour.*, July 10, 1897,) emphasises the difficulties met with in malrotated face cases. When the chin is to the front no other treatment than that employed in vertex cases will be needed; but when the chin is posterior, one should leave the case to nature till, after complete or nearly complete dilatation of the os externum and rupture of the membranes, one recognises that in spite of good pains the chin does not in an hour or two pass into the pelvic cavity. If, then, there are signs of danger in the lower uterine segment, and if the fetal heart-beats are diminishing in number and becoming irregular, the time has come for changing the presentation by "redressement." This treatment the author prefers to version, even when immediate delivery is necessary, and he follows it at once by the use of forceps. He thinks there is less danger in applying forceps to the occiput in its high position than in trying version when the lower uterine segment is much distended.

---

### VENTROFIXATION IN RETROVERSION OF THE UTERUS.

A. Lapthorn Smith, of Montreal, read a paper before the American Gynecological Society, at Washington, May 6, 1897, on this subject. His paper was based upon ninety-four ventrofixations and fifty-three Alexander's operations. He held that ventrofixation was the only operation that should be entertained in cases of retroversion with adhesions; but it should not be done when the uterus was movable and when there was no disease of the appendages requiring abdominal section, in which cases Alexander's operation had given excellent results. There should be no death-rate to either operation, neither should there ever be hernia, either ventral or inguinal, if the following directions were followed: the two operations were equally easy, although a few years ago the author was opposed to Alexander's operation on account of its difficulty. Now he could invariably find the ligaments, and generally in from half a minute to a minute and a half. He warned his hearers not to do Alexander's operation if there were any adhesions, even if they were loose enough to permit the uterus to be lifted up; because they would be put upon the stretch and would drag so much upon the ligaments as to finally pull them out of their anchorage. In laying down the technique of Alexander's operation he placed great stress upon the importance of putting aside all cutting instruments as soon as the skin, superficial and deep fascia had been cut through. Instead of laying open the inguinal canal, as advocated by some writers, he advised his hearers not to cut a single fiber of the intercolumnar fascia, which was the principal support of the pillars. Moreover, he said, the slightest nick of the fascia of the internal oblique would lead to a false passage and failure to find the ligament. If no cutting instruments were used, but only a Pean's forceps to draw out the ligament, there would be no difficulty in finding it, because there was nothing else in the canal but the ligament. In fact, with the eyes bandaged it could be found and drawn out, simply by introducing the closed forceps and then opening them, when the round ligament will fall into them and can be drawn out. He advocated the use of fine silkworm gut, which could be thoroughly sterilised and left in permanently. Occasionally he had been obliged to remove a buried stitch. In case any fibers of the intercolumnar or internal oblique should be accidently cut, great care should be exercised in sewing them up to avoid hernia. He had only one relapse after ventrofixation and one after Alexander, which were both subsequently repaired. Several of the cases of ventrofixation had since become pregnant and had had normal confinements. Also several cases of Alexander had had children. Many of the patients had been bed-ridden invalids for years before and were now enjoying excellent health. Both operations, each in its proper sphere, had given the greatest possible satisfaction.

